# Liver resection volume-dependent pancreatic strain following living donor hepatectomy

**DOI:** 10.1038/s41598-024-57431-1

**Published:** 2024-03-21

**Authors:** Taiichi Wakiya, Yasunaru Sakuma, Yasuharu Onishi, Yukihiro Sanada, Noriki Okada, Yuta Hirata, Toshio Horiuchi, Takahiko Omameuda, Kiichiro Takadera, Naohiro Sata

**Affiliations:** Division of Gastroenterological, General and Transplant Surgery, Department of Surgery, Jichi Medical University, 3311-1 Yakushiji, Shimotsuke, Tochigi 329-0498 Japan

**Keywords:** Gastrointestinal system, Hepatology, Risk factors

## Abstract

The liver and pancreas work together to recover homeostasis after hepatectomy. This study aimed to investigate the effect of liver resection volume on the pancreas. We collected clinical data from 336 living liver donors. They were categorized into left lateral sectionectomy (LLS), left lobectomy, and right lobectomy (RL) groups. Serum pancreatic enzymes were compared among the groups. Serum amylase values peaked on postoperative day (POD) 1. Though they quickly returned to preoperative levels on POD 3, 46% of cases showed abnormal values on POD 7 in the RL group. Serum lipase levels were highest at POD 7. Lipase values increased 5.7-fold on POD 7 in the RL group and 82% of cases showed abnormal values. The RL group’s lipase was twice that of the LLS group. A negative correlation existed between the remnant liver volume and amylase (r = − 0.326)/lipase (r = − 0.367) on POD 7. Furthermore, a significant correlation was observed between POD 7 serum bilirubin and amylase (r = 0.379)/lipase (r = 0.381) levels, indicating cooccurrence with liver and pancreatic strain. Pancreatic strain due to hepatectomy occurs in a resection/remnant liver volume-dependent manner. It would be beneficial to closely monitor pancreatic function in patients undergoing a major hepatectomy.

## Introduction

Maintenance of systemic homeostasis requires the coordination of multiple organs and tissues. To respond to multiple stress, higher organisms have developed a system of inter-organ communication, called inter-organ crosstalk^1–3^. Although the benefits outweigh the risks, surgery does put a strain on the body. The organs must work together to overcome the stress caused by surgery. Dysregulation of inter-organ coordination leads to new problems after surgery. In short, elucidation of inter-organ crosstalk is essential for comprehending the effects of surgery. However, these mechanisms are not fully understood.

The liver and pancreas are digestive organs that work cooperatively to maintain proper metabolic status. Thus, the dysfunction of one organ can affect the function of the other. Furthermore, they are anatomically connected by the portal system. Therefore, an organic change from surgery on one organ can have unfavorable effects on the other organ. For example, it has long been known that insulin hypersecretion from pancreatic islets occurs after a partial hepatectomy^4,5^. Furthermore, previous studies have shown hyperamylasaemia and pancreatitis after hepatectomy and liver transplantation (LT)^6–9^. Although most often hyperamylasaemia and pancreatitis can be subclinical, it can manifest as severe pancreatitis resulting in multiple organ failure, increasing morbidity and mortality following liver surgery^10,11^. Several lines of evidence from clinical studies have demonstrated that portal vein pressure increased after major hepatectomy in patients without cirrhosis^12–14^. Portal congestion following hepatectomy has been proposed as one of the pathophysiologic mechanisms responsible for pancreatic impairments^15,16^. However, the detailed mechanism of liver-pancreas crosstalk and associated risk factors remains unclear.

Given the potential role of portal congestion in pancreatic stress, it is imperative to understand how the resection volume of liver impacts on pancreas. Despite its importance and the existence of experimental studies^4,5^, no analysis has been conducted on human populations. This represents a significant gap in our understanding and underscores the urgent need for further investigation. Therefore, this study aimed to investigate the effect of liver resection volume on the pancreas. Here, by analyzing living liver donors, a cohort with relatively uniform liver and pancreatic function, we have demonstrated a significant relationship between liver resected volume and pancreatic burden.

## Methods

### Patients and study design

This single-center, retrospective, observational study was approved by the Ethics Committees of Jichi Medical University (Ethics Committee Approval Case Number 20-001). This study was designed and carried out in accordance with the Declaration of Helsinki and Istanbul. The need for written informed consent for the present study was waived by the Institutional Review Board of Jichi Medical University in view of its retrospective design, in accordance with national and local guidelines, such as the fact that all clinical/laboratory measurements and procedures were part of routine care.

A total of 339 living liver donors undergoing hepatectomy at our facility between 2003 and 2023 were included in this study. One case using grafts from donors with familial amyloid polyneuropathy was excluded. Additionally, to preserve the integrity and uniformity of the group comparisons, donors who had undergone posterior sectionectomy (n = 2) were also excluded. Finally, 336 consecutive cases were analyzed. Baseline clinical data were obtained from the medical records. In this study, plasma enzymatic activities of total amylase were analyzed.

### Surgical procedures and operative management

The type of donor hepatectomy was selected based on the recipient’s standard liver volume, weight, and graft volume determined by preoperative computed tomographic volumetry. The formula for calculating the standard liver volume is as follows: standard liver volume (mL) = 706.2 × body surface area (m^2^) + 2.4. To calculate the ratio of the remnant liver volume to the total liver volume, we used the following formula: (standard liver volume − resected volume)/standard liver volume. We performed a left lateral sectionectomy (LLS) for LLS grafts and an in vivo segment 3 (S3) resection during LLS for S2 monosegment grafts as previously reported^17^. The donor’s biliary anatomy was evaluated using intraoperative real-time cholangiography performed three times to determine the biliary anatomy, decide on the biliary transection line, and confirm absence of biliary leakage. A catheter was inserted into the bile duct via the cystic duct. Intraoperative cholangiography was initiated using C-arm fluoroscopy, and a water-soluble contrast agent was slowly injected through the catheter. A bulldog clamp was applied to the common bile duct to prevent retrograde flow during the procedure. Donor graft hepatectomy was routinely performed with intraoperative ultrasonographic guidance. In this cohort, we transected the liver parenchyma without blood inflow clamping such as the Pringle maneuver.

### Classification and comparison of patients

The 336 living donors were divided into three groups, based on the type of hepatectomy as follows: the left lateral sectionectomy (LLS) group, the left lobectomy (LL) group, and the right lobectomy (RL) group. Due to the similarity in remnant liver volume, the donors for monosegmentectomy graft (n = 17) and reduced LLS graft (n = 15) were included in the LLS group. In comparing perioperative factors, the medical records for each case were reviewed and compared among the groups.

### Statistical analyses

Continuous variables were expressed as the medians (ranges) and analyzed using nonparametric methods for non-normally distributed data (Mann–Whitney *U*-test). Categorical variables were reported as numbers (percentages) and analyzed using the chi-squared test or Fisher’s exact test, as appropriate. In multiple comparisons, results were assessed using the Kruskal–Wallis test with a Dunn–Bonferroni adjustment. The correlation between two parameters was analyzed by the Spearman rank-order method. Variables with a significant relationship to abnormal amylase/lipase values on postoperative day (POD) 7, in univariate analysis were used in a binary logistic regression model. A difference was considered to be significant for values of P < 0.05. Statistical analyses were performed using GraphPad Prism (v9.5.1; GraphPad Software, San Diego, CA, USA, https://www.graphpad.com) and JASP (v0.17.2; JASP team, Amsterdam, The Netherlands).

### Ethics approval and consent to participate

This study was approved by the Ethics Committees of Jichi Medical University (Ethics Committee Approval Case Number 20-001). Informed consent was obtained in the form of opt-out on our website (https://www.jichi.ac.jp/transplant/contents/disclosure.html), with the approval of the Ethics Committees of Jichi Medical University. The need for written informed consent for the present study was waived by the Institutional Review Board of Jichi Medical University in view of its retrospective design, in accordance with national and local guidelines, such as the fact that all clinical/laboratory measurements and procedures were part of routine care. Our study did not include minors. This study was designed and carried out in accordance with the Declaration of Helsinki and Istanbul.

## Results

### Comparison of the clinical characteristics and operation-related factors across the groups

First, we investigated the clinical characteristics and operative outcomes according to each of the procedures. Of the 336 donors, 208 (61.9%) were included in the LLS group, 89 (26.5%) in the LL group, and 39 (11.6%) in the RL group. A comparison of the clinical characteristics and operation-related factors between groups is shown in Table 1. There were significant differences in age and body mass index between groups. There were no significant differences in body surface area and calculated standard liver volume across the groups.

**Table 1:** Comparison of the clinical characteristics and operation-related factors in the entire cohort.

	All (n = 336)	RL (n = 39)	LL (n = 89)	LLS (n = 208)	P value	RL vs. LL P value	RL vs. LLS P value	LL vs. LLS P value
Gender, male, n	166 (49.1)	17 (43.6)	46 (51.7)	103 (49.5)	0.700			
Age, year	35 (20–67)	44 (24–63)	42 (20–56)	33 (23–67)	< 0.001	0.120	< 0.001	< 0.001
Body height, m	1.65 (1.46–1.93)	1.62 (1.52–1.86)	1.66 (1.48–1.93)	1.65 (1.46–1.84)	0.336			
Body weight, kg	60.0 (39.0–94.7)	60.4 (46.9–82.0)	62.6 (43.0–90.6)	58.8 (39.0–94.7)	0.086			
Body mass index, kg/m^2^	21.9 (17.3–31.4)	22.5 (18.6–28.3)	22.3 (17.3–30.8)	21.5 (17.5–31.4)	0.015	> 0.999	0.153	0.034
Body surface area, m^2^	1.65 (1.28–2.15)	1.65 (1.40–2.06)	1.69 (1.38–2.12)	1.64 (1.28–2.15)	0.166			
Standard liver volume, mL	1171 (908–1520)	1167 (994–1459)	1198 (980–1502)	1157 (908–1520)	0.166			
Preoperative AST, U/L	16 (10–51)	16 (12–33)	16 (11–51)	16 (10–46)	0.664			
Preoperative ALT, U/L	15 (5–65)	15 (8–41)	15 (5–65)	16 (6–62)	0.805			
Preoperative amylase, U/L	85 (22–300)	72 (43–169)	88 (43–239)	87 (22–300)	0.054			
Preoperative lipase, U/L	31 (12–64)	30 (18–61)	33 (19–64)	31 (12–62)	0.868			
Operative outcomes								
Operation time, min	327 (167–728)	397 (253–591)	367 (214–641)	293 (167–728)	< 0.001	0.114	< 0.001	< 0.001
Intraoperative blood loss, mL	530 (0–3050)	410 (100–1220)	800 (122–3050)	510 (0–2720)	< 0.001	< 0.001	0.208	< 0.001
Graft volume, g	260 (93–872)	630 (415–872)	363 (212–686)	224 (93–382)	< 0.001	< 0.001	< 0.001	< 0.001
Remnant liver volume ratio	0.77 (0.32–0.93)	0.47 (0.32–0.59)	0.70 (0.49–0.83)	0.81 (0.68–0.93)	< 0.001	< 0.001	< 0.001	< 0.001
Postoperative outcomes								
POD1 AST, U/L	340 (89–2010)	384 (146–1248)	361 (147–1397)	309 (89–2010)	0.211			
POD1 ALT, U/L	405 (82–1864)	399 (227–1238)	415 (189–1277)	385 (82–1864)	0.638			
POD1 total bilirubin, mg/dL	1.52 (0.58–7.07)	2.58 (1.27–5.33)	1.58 (0.58–7.07)	1.42 (0.59–6.13)	< 0.001	< 0.001	< 0.001	0.603
Postoperative hospital stay, day	10 (7–57)	11 (8–52)	10 (7–57)	10 (7–44)	0.083			

*AST* aspartate transaminase, *ALT* alanine transaminase, *LL* left lobectomy group, *LLS* left lateral sectionectomy group, *POD* postoperative day, *RL* right lobectomy group.

In the RL group, operation time was significantly longer compared to other groups. The LL group was significantly associated with a greater amount of intraoperative blood loss. Conversely, there were no significant differences in intraoperative bleeding between the RL group and the LLS group (P = 0.208). Consistently, actual graft volumes were significantly different between the groups (P < 0.001).

### Assessment of preoperative serum amylase and lipase values

**Figure 1:**
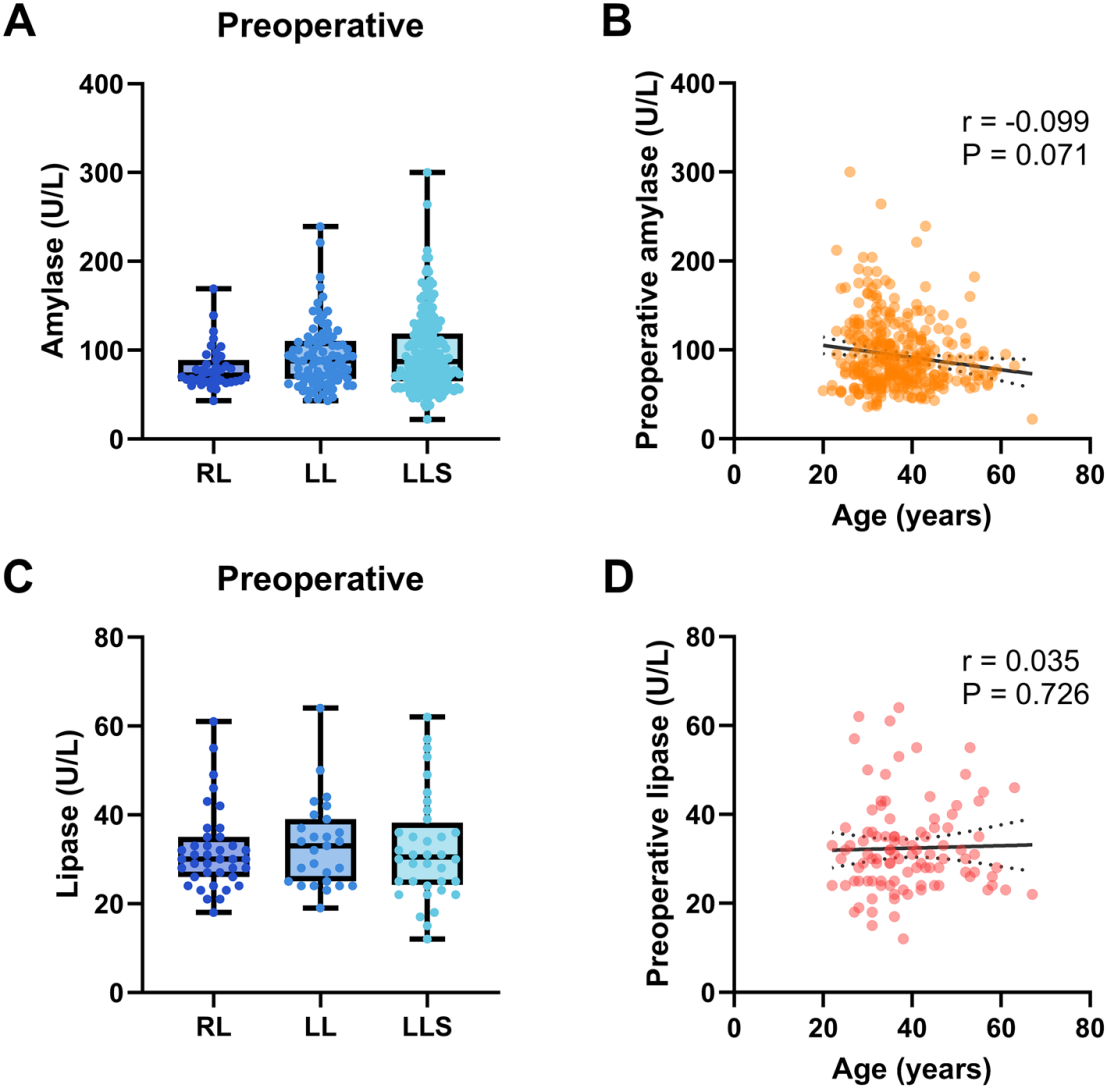
Assessment of preoperative serum amylase and lipase values. (**A**) Preoperative amylase levels of the donors. Data are shown as the median ± range. (**B**) Spearman rank correlation between preoperative amylase levels and age. (**C**) Preoperative lipase levels. (**D**) Spearman rank correlation between preoperative lipase levels and age. *LL* left lobectomy group, *LLS* left lateral sectionectomy group, *RL* right lobectomy group.

A comparison of the preoperative serum amylase and lipase during the perioperative period across the groups is shown in Fig. 1. There were no significant differences in the preoperative serum amylase levels among the groups (Fig. 1a). Because the LLS group was significantly younger than the RL group (Table 1), we analyzed the correlation between age and preoperative amylase levels. We found a slight negative correlation there (r = − 0.099, P = 0.071) (Fig. 1b). In contrast, there were no significant differences in the preoperative serum lipase levels across the groups (Fig. 1c). Moreover, there was no correlation between age and preoperative lipase levels (Fig. 1d).

### The RL group shows a more significant increase in amylase and lipase after surgery

**Figure 2:**
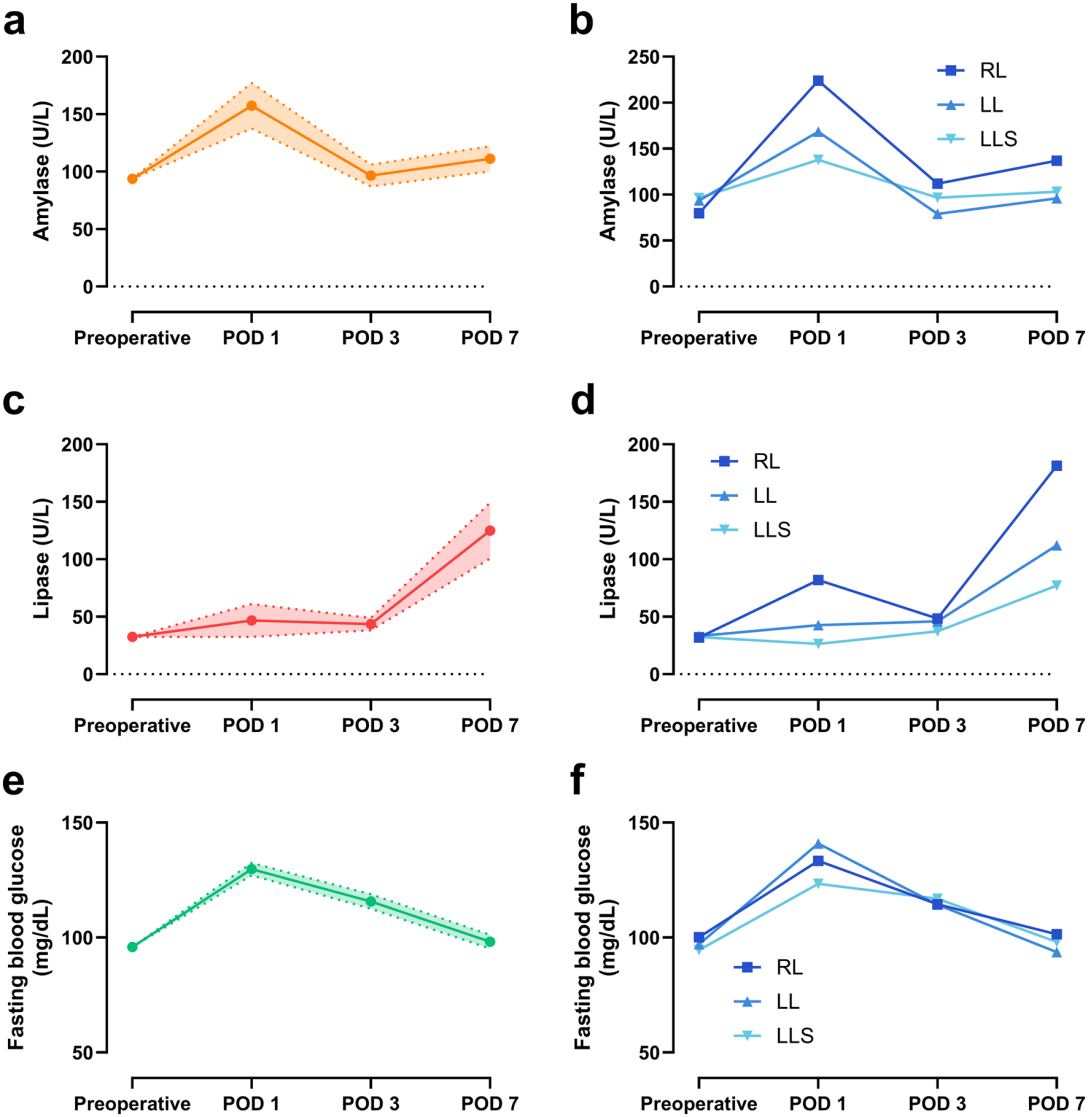
Chronological changes of serum amylase and lipase values during the perioperative period. (**a**) Amylase levels of the donors. Data are shown as the mean ± 95% confidence interval. (**b**) Chronological changes of perioperative amylase levels based on the procedure. (**c**) Perioperative lipase levels. (**d**) Chronological changes of perioperative lipase levels based on the procedure. (**d**) Perioperative fasting blood glucose levels. (**d**) Chronological changes of perioperative fasting blood glucose levels based on the procedure. *LL* left lobectomy group, *LLS* left lateral sectionectomy group, *POD* postoperative day, *RL* right lobectomy group.

Next, we clarified the chronological changes in amylase and lipase after hepatectomy (Fig. 2) and compared them across the groups (Supplemental Fig. 1). The amylase values peaked on the day after surgery. They quickly returned to preoperative levels on POD 3 (Fig. 2a). Postoperative amylase levels in the RL group were higher than those in other groups. The pattern of chronological change was similar for all three groups (Fig. 2b).

In contrast, the lipase increase on the day after hepatectomy was mild. Notably, the highest postoperative lipase levels were often found at POD 7. In the RL group, especially, there was an initial small increase, a decrease, and then a significant increase again (Fig. 2c). These results indicated the presence of a bimodal peak in the postoperative lipase dynamics of the RL group (Fig. 2d). These results also demonstrated different kinetics in serum amylase and lipase after hepatectomy (Supplemental Fig. 2).

Based on the differences in the preoperative amylase values, we further examined the relative values to the preoperative values. In the RL group, the amylase value increased 1.5-fold on POD 1 compared to those of before surgery (Supplemental Fig. 1). On POD 1, there was a significant difference between the RL group and the LLS group (P = 0.038). In the comparison of relative values, the higher values in the RL group were more remarkable than in the other groups. Likewise, in comparing relative values of lipase, the higher values in the RL group were more noticeable than in the other groups. Surprisingly, the lipase values increased 5.7-fold on POD 7 in the RL group. The RL groups showed a significant twofold increase compared to the LLS group (P < 0.001) (Supplemental Fig. 1).

We also elucidated the chronological changes in fasting blood glucose following hepatectomy. Regarding to the fasting blood glucose level, the peak postoperative levels were often observed on POD 1 (Fig. 2e). The pattern of chronological change was similar for all three groups (Fig. 2f). In contrast to the pancreatic exocrine enzymes, there was minimal variation in the blood glucose levels on POD 7 among the three groups.

### Effect of liver resection on postoperative lipase homeostasis may persist for several weeks

**Figure 3:**
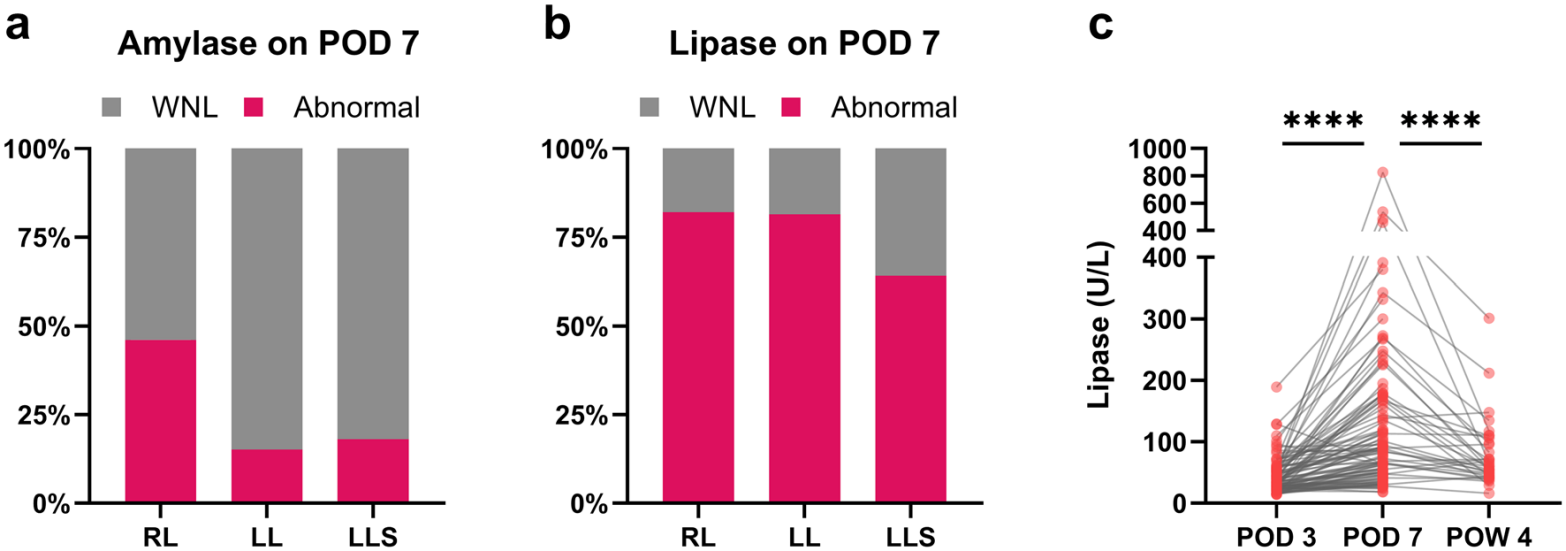
Percentage of abnormal values on POD 7. (**a**) Amylase, normal range 44–132 U/L. (**b**) lipase, normal range 13–49 U/L. (**c**) Paired dot plot of lipase values. Wilcoxon signed-rank test was performed (****P < 0.0001). *LL* left lobectomy group, *LLS* left lateral sectionectomy group, *POD* postoperative day, *POW* postoperative week, *RL* right lobectomy group, *WNL* within normal limits.

Next, we assessed the clinical significance of amylase and lipase values on POD 7. Figure 3 shows the percentage of cases with abnormal values in the measurements on POD 7. In the measurement of amylase, abnormal values are found in approximately 25% of the cases (34/133). In the RL group, 46% of cases showed abnormal values. Regarding lipase, approximately 75% of the cases (79/105) exhibited abnormal values on POD 7. Abnormal values were observed in more than 80% of cases after right or left lobectomy. These observations suggested that the effect of liver resection on postoperative lipase homeostasis may persist for several weeks. Therefore, although we were not able to evaluate all the cases, we assessed lipase levels at approximately four weeks after surgery. Consequently, many of the cases showed a trend toward improvement at one month after surgery (Fig. 3c).

### Negative correlation between the remnant liver volume and pancreatic strain

**Figure 4:**
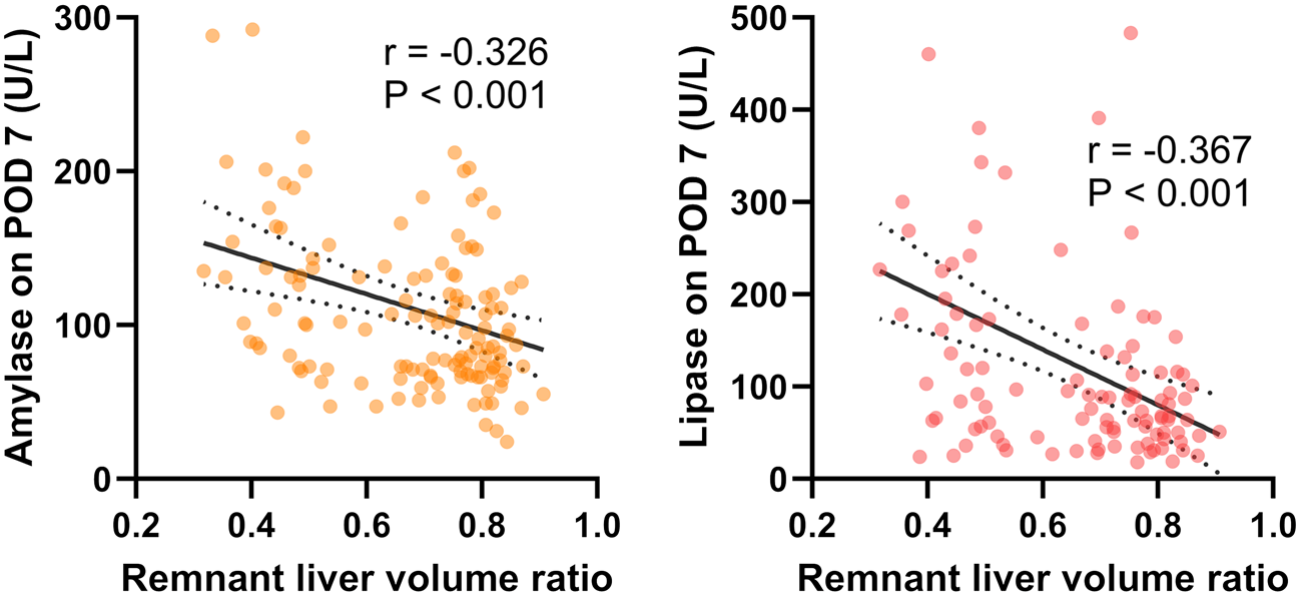
Correlation between the remnant liver volume and the pancreatic function test on postoperative day 7. This analysis excluded the cases with a monosegment graft and a reduced left lateral segment graft. In each scatter plot, two outliers are outside the axis limits. *POD* postoperative day.

The above data suggest a greater effect on pancreatic homeostasis in the RL group with larger resected liver volume. To clarify the relationship between the remnant liver volume and pancreatic strain, we performed a correlation test. As a result, we found a significant moderate negative correlation among them (Fig. 4).

### Risk factor for abnormal amylase and lipase values on POD 7

To seek the risk factor for the abnormal values on POD 7, we performed a binary logistic regression analysis. Significant variables linked with the abnormal lipase levels, which were found through a univariate analysis (P < 0.05), as listed in Supplemental Table 1, were entered into a binary regression analysis. Binary logistic regression indicated that the preoperative lipase value was a significant predictor of the abnormal lipase value on POD 7 (Wald = 8.019, P = 0.005). The odds ratio (OR) was 1.132 (95% confidence interval [CI]: 1.039–1.233). Regarding the abnormal amylase values on POD 7, the remnant liver volume ratio was significant at the 5% level (Wald = 11.019, P < 0.001). The OR was 5.554 × 10–4 (95% CI: 0.000–0.046) (Supplemental Table 2). Collectively, these results suggest that the greater the amount of liver resected, the greater the impact on the pancreas.

### No synergistic effect of aging on the amylase and lipase values on POD 7

**Figure 5:**
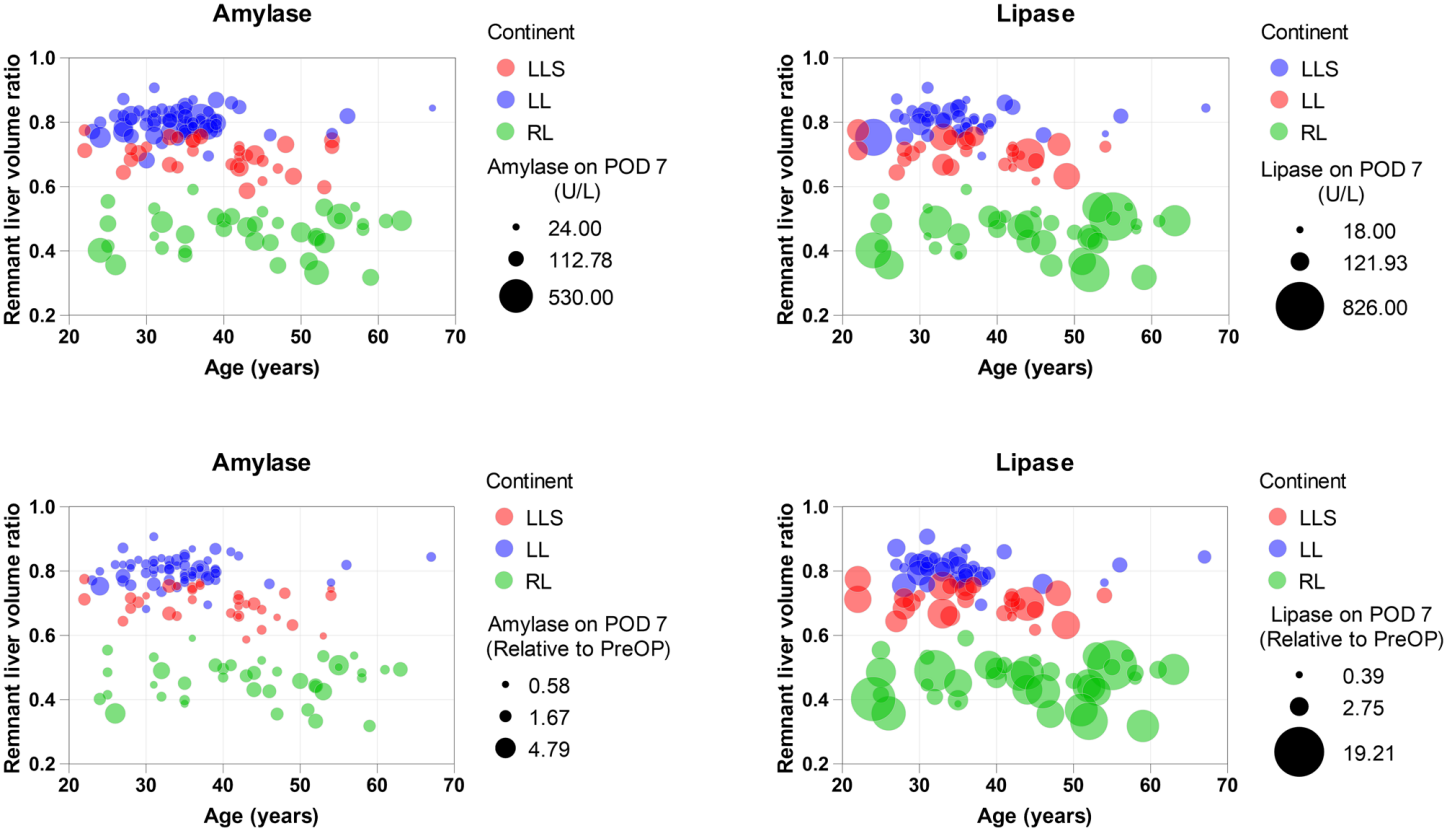
Bubble plot consisting of remnant liver volume ratio (Y axis), age (X axis), pancreatic enzyme levels on postoperative day 7 (bubble size based on values), and graft type (categorized by color). This analysis excluded the cases with a monosegment graft and a reduced left lateral segment graft. The top panels are bubbles of measurement values and the bottom panels are bubbles of values relative to preoperative values. *LL* left lobectomy group, *LLS* left lateral sectionectomy group, *POD* postoperative day, *RL* right lobectomy group.

We examined whether the effect of resection volume on pancreatic enzyme levels varied with age. A bubble plot consisting of remnant liver volume, age, pancreatic enzyme levels on POD 7, and graft type is shown in Fig. 5. In addition, we analyzed the correlation between age and pancreatic function tests on POD 7 in the RL group (Supplemental Fig. 3). In the present cohort, the synergistic effect of aging was not obvious.

### Implications from the correlation between postoperative liver and pancreatic strain

**Figure 6:**
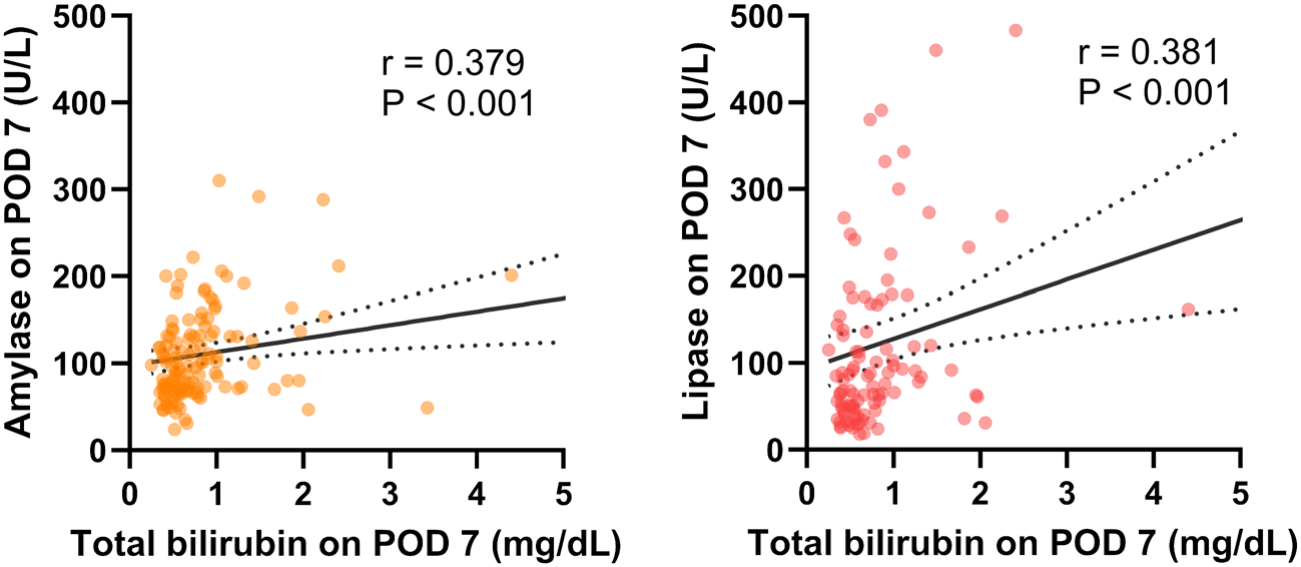
Correlation between the serum bilirubin levels and the pancreatic function test on postoperative day 7. This analysis excluded cases with a monosegment graft and a reduced left lateral segment graft. In each scatter plot, two outliers are outside the axis limits. *POD* postoperative day.

To draw a definitive conclusion, direct evidence of the relationship between pancreatic strain and portal vein pressure after hepatectomy would be ideal. However, continuous measurement of portal vein pressure during the perioperative period was not ethically feasible in living donors. Portal venous pressure increase after hepatectomy has recently been documented to correlate with liver dysfunction^13^. Thus, we assessed the association between liver and pancreas strain following hepatectomy. We examined the correlation between serum bilirubin levels, a representative marker of postoperative liver dysfunction, on POD 7 and amylase levels on POD 7. A significant positive correlation was observed (r = 0.379, P < 0.001). Similarly, there was a significant positive correlation between serum bilirubin levels on POD 7 and lipase levels on POD 7 (r = 0.381, P < 0.001) (Fig. 6). In summary, our findings underscore the link between postoperative liver and pancreatic strain. These results indicate that clinicians need to be aware of the potential for pancreatic strain in patients showing signs of postoperative liver dysfunction.

### Correlation between posthepatectomy complications and pancreatic strain

We further evaluated the clinical implications of postoperative increases in serum amylase and lipase. In this study, three donors (3/336, 0.9%) were diagnosed with mild acute pancreatitis based on the Atlanta classification and definitions by international consensus^18^. These three donors had all undergone a right lobectomy. In short, the incidence of postoperative pancreatitis in the RL group was 7.7% (3/39).

We compared the postoperative complications rates according to lipase levels on POD 7 in three groups (less than 49 U/L vs. 50–147 U/L vs. 148 U/L or more). Although the duration of postoperative hospital days was longer in the highest group, there were no significant differences in postoperative complications among the groups (Supplemental Table 3). Similar results were obtained in an analysis stratifying the amylase levels on POD 7 into three groups (Supplemental Table 4).

## Discussion

We have investigated liver-pancreas crosstalk after hepatectomy. Our study demonstrated that the impact on the pancreas was resection/remnant liver volume-dependent. Interestingly, we also found different kinetics in serum amylase and lipase after living donor hepatectomy.

Previous studies have shown various pancreatic reactions associated with liver surgery^4–11^. Most of the studies reported subclinical hyperamylasaemia, but there were some reports of clinically relevant pancreatitis. The detailed mechanism still remains unclear. One possible explanation for these reactions could be an ischemic reperfusion injury (IRI). In general, IRI occurs in LT or hepatectomy under a temporary vascular clamp for hemorrhage control. The spillage of cytokines and reactive oxygen species from the liver has been implicated in the pathogenesis of pancreatic injury^19–22^. Another possible explanation could be portal congestion^15,16^. Theoretically, portal vein congestion related to liver surgery is divided into two types: intraoperative congestion, associated with surgical manipulation such as a vascular clamp, and postoperative transitional congestion. Miyagawa et al. reported that the use of the Pringle maneuver increased the incidence of postoperative hyperamylasemia. They also found that the degree of hyperamylasemia was increased by portal congestion with prolonged vascular occlusion time^15^. Following hepatectomy, diverting the whole portal flow to the remnant liver causes an increase in portal venous pressure^23,24^. This transitional portal venous overpressure can lead to postoperative liver failure as well as pancreatic vein congestion. Such congestion has been suggested as a key pathophysiological mechanism behind pancreatic impairments^25,26^. Moreover, recent studies have shown that the hemodynamic changes resulting from pancreatic congestion could impair pancreatic acinar cells^27,28^. In the present study, as institutional policy, we routinely performed hepatectomy without a vascular clamp. Therefore, it is unlikely that IRI and intraoperative portal vein congestion were the direct main cause of stress to the pancreas in this cohort. Taken together, these data suggest that the impact of transitional portal venous overpressure was significant. The key result of our study, in which pancreatic enzymes became elevated in a resection/remnant liver volume-dependent manner, supports this interpretation.

Previous research has highlighted age-related pathological changes in the pancreas^29–32^, suggesting that variables such as age and the underlying state of the pancreas can affect its functionality and stress responses following hepatectomy. Similarly, our study found a slight negative correlation between age and preoperative amylase levels. Yet, the link between postoperative responses and age was not clear-cut. The influence of individual donor characteristics on pancreatic responses after hepatectomy calls for more detailed exploration in future studies.

How does pancreatic damage/due to hepatectomy affect the remnant liver? Several in vitro and in vivo studies demonstrated insulin hypersecretion after hepatectomy^4,5^. The liver plays a central role in the control of glucose homeostasis collaborating with insulin and other hormones^33–35^. Thus, insulin hypersecretion after hepatectomy is thought to be a reasonable adaptation physiologically. Paradoxically, pancreatic impairment interferes with insulin hypersecretion leading to disruption of blood glucose homeostasis, resulting in unfavorable postoperative outcomes including liver dysfunction^36–38^. More importantly, insulin, a representative anabolic hormone, is essential for liver regeneration^39–41^. Collectively, we need to recognize that serious damage to the pancreas may have a negative impact on liver regeneration after hepatectomy.

We explored a correlation between postoperative liver and pancreatic strain. Severe liver dysfunction after hepatectomy is recognized as posthepatectomy liver failure (PHLF). The incidence of PHLF fluctuates based on the diagnostic criteria employed and is presently estimated to be 3–28%^42–45^. PHLF continues to be the leading cause of postoperative mortality and poses a substantial clinical challenge for the hepatobiliary surgeon. Once PHLF develops, options are often limited to best supportive care. Needless to say, the optimal strategy is to reduce the risk of PHLF occurrence. In this study, two donors were categorized as grade A for PHLF as defined by the International Study Group of Liver Surgery^46^. Unfortunately, pancreatic enzymes levels were not measured in one of the donors. However, in the other, the lipase level exceeded three times the upper limit of normal on POD 7 (227 U/L), and the patient also had persistent upper abdominal pain indicative of pancreatitis, thus meeting the diagnostic criteria for acute pancreatitis. Our findings emphasize the necessity of recognizing the potential for concomitant pancreatic dysfunction in patients with PHLF. Additionally, they indicate the importance of offering optimal supportive care not only for the liver but also for the pancreas in patients with PHLF. Determining effective pancreatic intervention for PHLF is a topic that warrants further exploration.

Our study demonstrated novel insight into the underlying relationship between a resection/remnant liver volume and postoperative amylase/lipase dynamics; however, there are several limitations. First, this is a retrospective single-institution cohort study. Additionally, though we found hyperamylasemia and hyperlipasemia after hepatectomy for living liver donors, we have not yet clarified the involvement of pancreatic hormones such as glucagon and insulin. Needless to say, pancreatic hormones and cytokines need to be evaluated to elucidate liver-pancreas crosstalk following hepatectomy. We also acknowledge the potential utility of fecal enzyme measurements for a more accurate assessment of pancreatic exocrine function. While these measurements were not incorporated into our current study, including them in future research could offer a more thorough exploration of this aspect. Finally, we evaluated the total amylase activity, including salivary and pancreatic amylases. Any type of surgery under general anesthesia often increase salivary amylase, which is probably caused by tracheal intubation. Ideally, we should have measured pancreatic amylase, but it was not feasible. Thus, serum lipase was measured for a more pancreas-specific evaluation. Interestingly, our results demonstrated different kinetics in serum amylase and lipase after living donor hepatectomy. The different courses are difficult to explain by differences in half-life alone^47^. Possible reasons include, firstly, that lipase may specifically detect even minor pancreatic stress undetectable by amylase^47^. Secondly, after hepatectomy, the homeostasis and response of these two enzymes may differ due to changes in blood dynamics, metabolic responses, and liver regeneration^33,48^. This could indicate different adaptations to glucose and lipid metabolism after hepatectomy. Such result evokes the notion of a heterogeneity of stress and its adaptation in different pancreatic cells and tissues. This finding could serve as a foundation for future research in pancreatic biology, suggesting potential avenues for exploring the specific roles and responses of pancreatic enzymes following surgical interventions. Until this point is clarified, extensive postoperative evaluation, including monitoring various pancreatic functions, would be better, at least in high-risk cases.

In conclusion, we specifically examined the association between liver resection volume and pancreatic function. Our study indicated pancreatic strain due to hepatectomy occurs in a resection/remnant liver volume-dependent manner. Our findings emphasize the need to recognize the potential for concomitant pancreatic dysfunction in patients with PHLF. It would be advisable to closely monitor the pancreatic function of patients who have undergone a major hepatectomy, particularly in the initial postoperative month. Understanding liver-pancreas crosstalk following liver resection is essential for the success of hepatectomy in all cases. We expect that future studies will expand our understanding of the heterogeneity and whether different pancreatic cells/tissues have different adaptations to hepatectomy.

### Supplementary Information

Supplementary Information.

## Data Availability

The data generated or analyzed during this study are included in this published article and its supplementary information files.
