# Supplementary material for: Liver resection volume-dependent pancreatic strain following living donor hepatectomy
Source: Sci Rep. 2024 Mar 21;14:6753. doi: 10.1038/s41598-024-57431-1 (PMC10957952; doi:10.1038/s41598-024-57431-1)
Supplement: Supplementary file 1 — Supplementary Information. [file 41598_2024_57431_MOESM1_ESM.pdf]

# Liver Resection Volume-Dependent Pancreatic Strain Following Living Donor Hepatectomy

Taiichi Wakiya*, Yasunaru Sakuma, Yasuharu Onishi, Yukihiro Sanada, Noriki Okada, Yudai Hirata, Toshio Horiuchi, Takahiko Omameuda, Kiichiro Takadera, Naohiro Sata.
*Corresponding author:

**Supplementary information**

Supplemental Content 1: Supplemental Figure 1, Assessment of serum amylase and lipase values after hepatectomy.
Supplemental Content 2: Supplemental Figure 2, Postoperative levels of amylase and lipase compared to their levels before surgery.
Supplemental Content 3: Supplemental Figure 3, Correlation between age and the pancreatic function test on postoperative day 7.
Supplemental Content 4: Supplemental Table 1, Comparison between groups with normal and abnormal lipase levels on POD 7.
Supplemental Content 5: Supplemental Table 2, Comparison between groups with normal and abnormal amylase levels on POD 7.
Supplemental Content 6: Supplemental Table 3, Comparison of postoperative complications rates according to lipase levels on POD 7.
Supplemental Content 7: Supplemental Table 4, Comparison of postoperative complications rates according to amylase levels on POD 7.

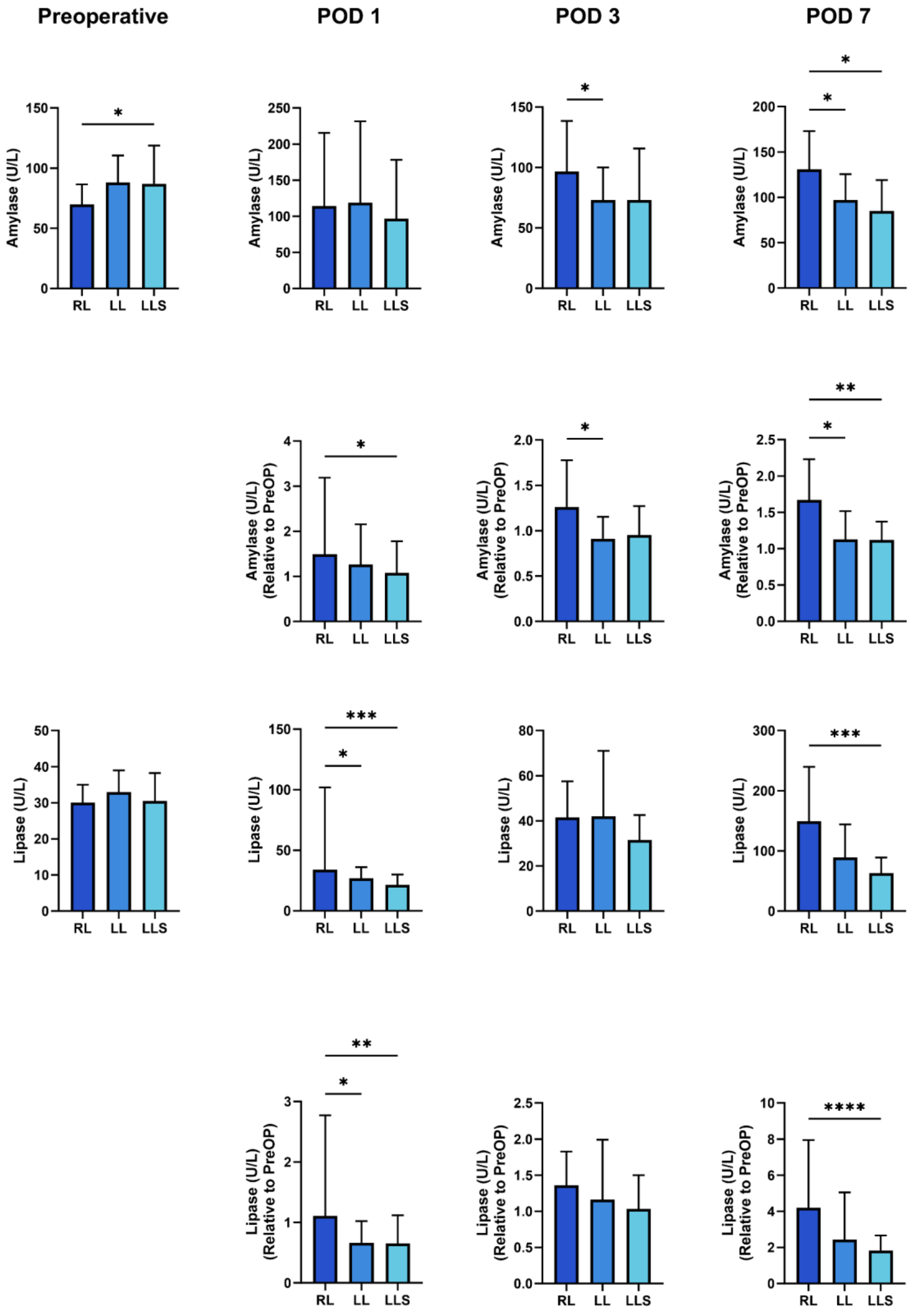


**Supplemental Figure 1.**
Assessment of serum amylase and lipase values after hepatectomy. Data are shown as the median ± interquartile range (*P < 0.05, **P < 0.01, ***P < 0.001, ****P < 0.0001). LL, left lobectomy group; LLS, left lateral sectionectomy group; POD, postoperative day; PreOP, preoperative value; RL, right lobectomy group.

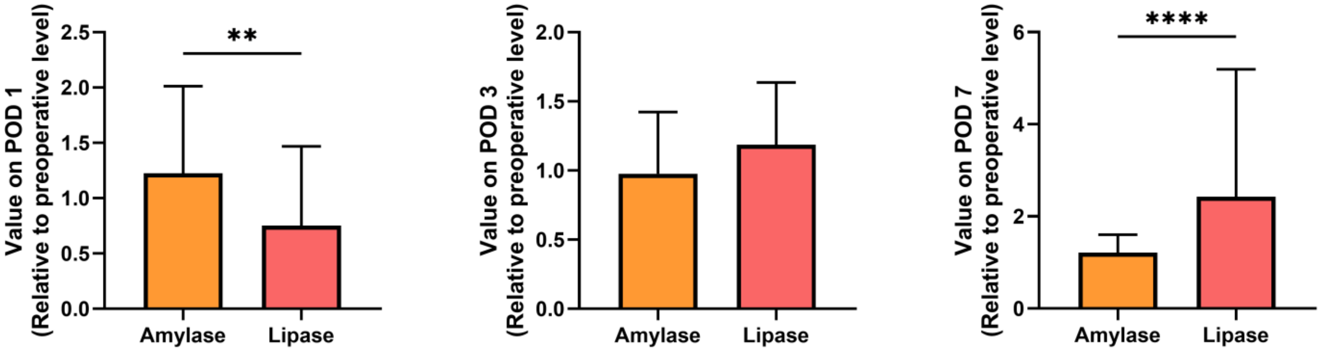


**Supplemental Figure 2.**

Postoperative levels of amylase and lipase compared to their levels before surgery. Data are shown as the median ± interquatile range (**P < 0.01, ****P < 0.0001). POD, postoperative day.

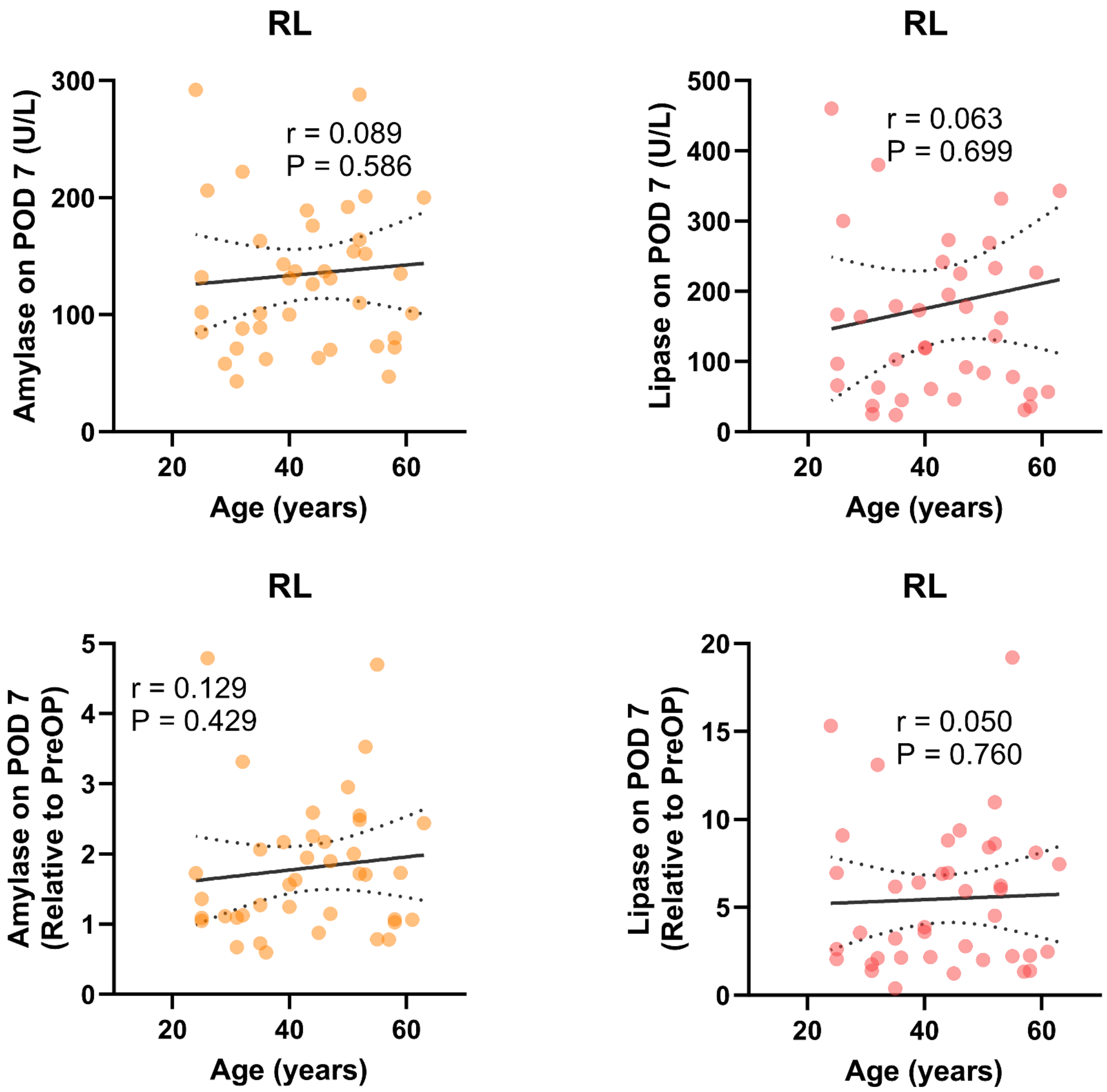


**Supplemental Figure 3.**

Correlation between age and the pancreatic function test on postoperative day 7 in the RL group. The top panel is a plot of measured values and the bottom panel is a plot of values relative to preoperative values. POD, postoperative day; PreOP, preoperative value; RL, right lobectomy group.

**Supplemental Table 1: Comparison between groups with normal and abnormal lipase levels on POD 7**

| | POD 7 WNL (n = 26) | POD 7 Abnormal (n = 79) | P value | Logistic regression | | |
|---|---|---|---|---|---|---|
| | | | | Odds ratio | 95% CI | P value |
| Gender, male, n | 11 (42.3) | 37 (46.8) | 0.688 | | | |
| Age, year | 38 (27-67) | 36 (22-63) | 0.295 | | | |
| Body height, m | 1.62 (1.52-1.82) | 1.64 (1.51-1.88) | 0.651 | | | |
| Body weight, kg | 60.2 (44.9-94.7) | 59.0 (42.0-92.3) | 0.427 | | | |
| Body mass index, $kg/m^2$ | 23.2 (18.9-29.1) | 22.0 (17.6-29.2) | 0.074 | | | |
| Body surface area, $m^2$ | 1.64 (1.39-2.15) | 1.64 (1.36-2.12) | 0.755 | | | |
| Standard liver volume, mL | 1160 (987-1520) | 1162 (963-1502) | 0.764 | | | |
| Preoperative AST, U/L | 16 (12-46) | 16 (11-46) | 0.714 | | | |
| Preoperative ALT, U/L | 15 (6-41) | 14 (6-62) | 0.893 | | | |
| Preoperative amylase, U/L | 67 (22-139) | 76 (43-169) | 0.042 | 0.995 | 0.967-1.025 | 0.758 |
| Preoperative lipase, U/L | 24 (12-61) | 32 (19-64) | <0.001 | 1.132 | 1.039-1.233 | 0.005 |
| *Operative outcomes* | | | | | | |
| Operation time, min | 370 (244-518) | 362 (222-591) | 0.897 | | | |
| Intraoperative blood loss, mL | 350 (100-1560) | 330 (0-1230) | 0.518 | | | |
| Graft volume, g | 315 (146-872) | 379 (125-863) | 0.157 | | | |

| | | | | | | |
|---|---|---|---|---|---|---|
| Graft type, n | | | 0.127 | | | |
| LLS | 14 (53.8) | 25 (31.3) | | | | |
| LL | 5 (19.2) | 22 (27.5) | | | | |
| RL | 7 (26.9) | 32 (40.5) | | | | |
| Remnant liver volume ratio | 0.74 (0.39-0.87) | 0.70 (0.32-0.91) | 0.091 | | | |
| *Postoperative outcomes* | | | | | | |
| POD1 AST, U/L | 331 (147-856) | 327 (89-1433) | 0.908 | | | |
| POD1 ALT, U/L | 365 (152-1216) | 356 (126-1277) | 0.956 | | | |
| POD1 total bilirubin, mg/dL | 1.23 (0.75-3.62) | 1.91 (0.58-6.13) | 0.005 | 1.587 | 0.880-3.356 | 0.113 |
| POD1 amylase, U/L | 63 (26-1343) | 95 (26-1147) | 0.019 | 0.999 | 0.997-1.002 | 0.653 |
| POD1 lipase, U/L | 19 (10-108) | 30 (10-808) | 0.022 | 1.009 | 0.995-1.023 | 0.230 |

AST, aspartate transaminase; ALT, alanine transaminase; LL, left lobectomy group; LLS, left lateral sectionectomy group; POD, postoperative day; RL, right lobectomy group; WNL, within normal limits.

**Supplemental Table 2: Comparison between groups with normal and abnormal amylase levels on POD 7**

| | POD 7<br>WNL (n = 99) | POD 7<br>Abnormal (n = 34) | P value | Logistic regression | | |
|---|---|---|---|---|---|---|
| | | | | Odds ratio | 95% CI | P value |
| Gender, male, n | 46 (46.5) | 17 (50.0) | 0.722 | | | |
| Age, year | 35 (22-67) | 40 (24-63) | 0.069 | | | |
| Body height, m | 1.64 (1.49-1.93) | 1.66 (1.51-1.82) | 0.879 | | | |
| Body weight, kg | 60.4 (42.0-94.7) | 57.4 (45.0-90.6) | 0.096 | | | |
| Body mass index, kg/m$^2$ | 22.4 (17.6-29.2) | 20.8 (17.8-27.6) | 0.025 | 0.850 | 0.658-1.099 | 0.215 |
| Body surface area, m$^2$ | 1.67 (1.36-2.15) | 1.63 (1.40-2.12) | 0.242 | | | |
| Standard liver volume, mL | 1182 (963-1502) | 1154 (993-1502) | 0.247 | | | |
| Preoperative AST, U/L | 16 (11-46) | 17 (10-33) | 0.278 | | | |
| Preoperative ALT, U/L | 14 (6-62) | 16 (6-37) | 0.515 | | | |
| Preoperative amylase, U/L | 73 (22-182) | 84 (43-169) | 0.028 | 0.997 | 0.972-1.023 | 0.836 |
| Preoperative lipase, U/L | 30 (12-64) | 33 (24-55) | 0.086 | | | |
| *Operative outcomes* | | | | | | |
| Operation time, min | 342 (167-591) | 370 (173-495) | 0.418 | | | |
| Intraoperative blood loss, mL | 380 (0-3050) | 400 (100-1650) | 0.682 | | | |

| | | | | | | |
|---|---|---|---|---|---|---|
| Graft volume, g | 296 (125-872) | 532 (215-816) | 0.001 | † | | |
| Graft type, n | | | 0.002 | † | | |
| LLS | 50 (50.0) | 11 (32.3) | | | | |
| LL | 28 (28.0) | 5 (14.7) | | | | |
| RL | 21 (21.2) | 18 (52.9) | | | | |
| Remnant liver volume ratio | 0.75 (0.36-0.91) | 0.52 (0.32-0.82) | <0.001 | 5.554 x 10-4 | 0.000-0.046 | <0.001 |
| *Postoperative outcomes* | | | | | | |
| POD1 AST, U/L | 326 (89-1433) | 322 (112-1543) | 0.396 | | | |
| POD1 ALT, U/L | 380 (118-1268) | 365 (139-1645) | 0.503 | | | |
| POD1 total bilirubin, mg/dL | 1.49 (0.58-4.97) | 2.31 (0.85-6.13) | 0.001 | 1.116 | 0.617-2.020 | 0.716 |
| POD1 amylase, U/L | 73 (26-1343) | 126 (43-896) | <0.001 | 1.000 | 0.998-1.003 | 0.822 |
| POD1 lipase, U/L | 25 (10-239) | 40 (13-808) | 0.007 | 1.004 | 0.995-1.014 | 0.397 |

AST, aspartate transaminase; ALT, alanine transaminase; LL, left lobectomy group; LLS, left lateral sectionectomy group; POD, postoperative day; RL, right lobectomy group; WNL, within normal limits.

† Excluded due to multicollinearity with graft volume.

**Supplemental Table 3: Comparison of postoperative complications rates according to lipase levels on POD 7**

| reference range: 13-49 U/L | -49 U/L (n = 26) | 50-147 U/L (n = 51) | 148- U/L (n = 28) | P value |
|---|---|---|---|---|
| Posthepatectomy liver failure†, n | 0 | 0 | 0 | >0.999 |
| Bile leakage, n | 2 (7.7) | 8 (15.7) | 1 (3.6) | 0.211 |
| Delayed gastric emptying, n | 0 | 5 (9.8) | 0 | 0.062 |
| Wound infection, n | 1 (3.8) | 0 | 0 | 0.216 |
| Acute pancreatitis‡, n | 0 | 0 | 3 (10.7) | 0.014 |
| Postoperative hospital stay, day | 10 (7-19) | 10 (7-23) | 12 (8-52) | 0.016 |

POD, postoperative day

Lipase reference range: 13-49 U/L

† Based on the International Study Group of Liver Surgery definition.

‡ Based on the Atlanta classification and definitions by international consensus.

**Supplemental Table 4: Comparison of postoperative complications rates according to amylase levels on POD 7**

| | -132 U/L (n = 99) | 133-396 U/L (n = 33) | 397- U/L (n = 1) | P value |
|---|---|---|---|---|
| Posthepatectomy liver failure†, n | 1 (1.0) | 0 | 0 | 0.841 |
| Bile leakage, n | 13 (13.1) | 2 (6.1) | 0 | 0.505 |
| Delayed gastric emptying, n | 6 (6.0) | 1 (3.0) | 0 | 0.774 |
| Wound infection, n | 3 (3.0) | 0 | 0 | 0.590 |
| Acute pancreatitis‡, n | 0 | 3 (9.1) | 0 | 0.010 |
| Postoperative hospital stay, day | 10 (7-23) | 12 (8-52) | 16 | NA |

POD, postoperative day

Amylase reference range: 44-132 U/L

† Based on the International Study Group of Liver Surgery definition.

‡ Based on the Atlanta classification and definitions by international consensus.
